# Understanding the processes underpinning IMPlementing IMProved Asthma self-management as RouTine (IMP^2^ART) in primary care: study protocol for a process evaluation within a cluster randomised controlled implementation trial

**DOI:** 10.1186/s13063-024-08179-6

**Published:** 2024-06-04

**Authors:** J. Sheringham, L. Steed, K. McClatchey, B. Delaney, A. Barat, V. Hammersley, V. Marsh, N. J. Fulop, S. J. C. Taylor, H. Pinnock

**Affiliations:** 1 Institute of Epidemiology and Health Care, UCL, London, WC1E 6BT UK; 2 Wolfson Institute of Population Health, Queen Mary University of London, London, UK; 3 University of Dundee, Dundee, UK; 4 School of Health and Related Research, The University of Sheffield, Sheffield, UK; 5 Asthma UK Centre for Applied Research, Usher Institute, University of Edinburgh, Edinburgh, UK

**Keywords:** Asthma, Process evaluation, Implementation, Primary care, Self-management, IMP^2^ART

## Abstract

**Background:**

Providing supported self-management for people with asthma can reduce the burden on patients, health services and wider society. Implementation, however, remains poor in routine clinical practice. IMPlementing IMProved Asthma self-management as RouTine (IMP^2^ART) is a UK-wide cluster randomised implementation trial that aims to test the impact of a whole-systems implementation strategy, embedding supported asthma self-management in primary care compared with usual care. To maximise opportunities for sustainable implementation beyond the trial, it is necessary to understand how and why the IMP^2^ART trial achieved its clinical and implementation outcomes.

**Methods:**

A mixed-methods process evaluation nested within the IMP^2^ART trial will be undertaken to understand how supported self-management was implemented (or not) by primary care practices, to aid interpretation of trial findings and to inform scaling up and sustainability. Data and analysis strategies have been informed by mid-range and programme-level theory. Quantitative data will be collected across all practices to describe practice context, IMP^2^ART delivery (including fidelity and adaption) and practice response. Case studies undertaken in three to six sites, supplemented by additional interviews with practice staff and stakeholders, will be undertaken to gain an in-depth understanding of the interaction of practice context, delivery, and response. Synthesis, informed by theory, will combine analyses of both qualitative and quantitative data. Finally, implications for the scale up of asthma self-management implementation strategies to other practices in the UK will be explored through workshops with stakeholders.

**Discussion:**

This mixed-methods, theoretically informed, process evaluation seeks to provide insights into the delivery and response to a whole-systems approach to the implementation of supported self-management in asthma care in primary care. It is underway at a time of significant change in primary care in the UK. The methods have, therefore, been developed to be adaptable to this changing context and to capture the impact of these changes on the delivery and response to research and implementation processes.

**Supplementary Information:**

The online version contains supplementary material available at 10.1186/s13063-024-08179-6.

## Background

Asthma places a major burden on patients, health services and wider society. Providing self-management education to people with asthma, supported by a personalised action plan and regular review, can reduce this burden, by preventing unscheduled healthcare use and improving asthma control [1]. Several studies have demonstrated that the implementation of supported self-management in primary care, however, remains low [2]. Enhancing the implementation of supported self-management in primary care requires a whole-systems approach—i.e. a combination of patient education, professional training and organisational support [3].

IMP^2^ART is a whole-systems, evidence-based implementation strategy developed to help primary care practices to implement supported self-management for asthma patients [4–8]. Evaluations of such implementation strategies are complex and require consideration of the clinical effectiveness, the implementation success and the process by which such outcomes are achieved. Process evaluations play a particularly crucial role in this understanding. They unpick the ‘black box’ of interventions by understanding who received what, how and the process through which it impacted (or not) outcomes and inform potential mechanisms for sustainability. There is plentiful guidance that process evaluations should use mid-range theory in their design and delivery [9]. There is increasing recognition that process evaluations could and should seek to develop these mid-range theories to improve the design and evaluation of future implementation studies [10].

This paper describes the protocol for the process evaluation taking place alongside a cluster randomised trial of the IMPlementing IMProved Asthma self-management as RouTine (IMP^2^ART) strategy in UK primary care practices [ref: ISRCTN15448074]. It sets out how we seek to measure the delivery and response to IMP^2^ART, how we seek to understand the trial’s effectiveness findings and how it may contribute to the development of theory.

### IMP^2^ART cluster randomised trial

IMP^2^ART is a UK-wide cluster randomised implementation trial that aims to test the impact of a whole-systems implementation strategy embedding supported asthma self-management in primary care compared with usual care on both clinical and implementation outcomes. The main trial protocol and the IMP^2^ART strategy are described in McClatchey et al. [5].

#### Programme theory

IMP^2^ART combined the mid-range implementation and behaviour change theories of iPARIHS [9] and capability, opportunity, and motivation required for behaviour change (COM-B) [11] to develop a programme-level theory of how IMP^2^ART can support practices to implement supported self-management in asthma [12]. The programme theory states the central hypothesis of IMP^2^ART, i.e. that facilitation plays a critical role in implementation success. Facilitation encompasses both the inputs of a trained facilitator and the delivery of IMP^2^ART implementation strategies. Facilitation is expected to achieve its impacts through increasing staff capability, motivation and opportunity towards supported self-management. It is also expected that tailoring by facilitators to take account of practice context, particularly capacity, culture and leadership will be an important aspect of their interactions with practices. Moreover, we expect that the relationship between the facilitation and practice response to IMP^2^ART will be influenced by practice context.

### IMP^2^ART strategies

**Fig. 1:**
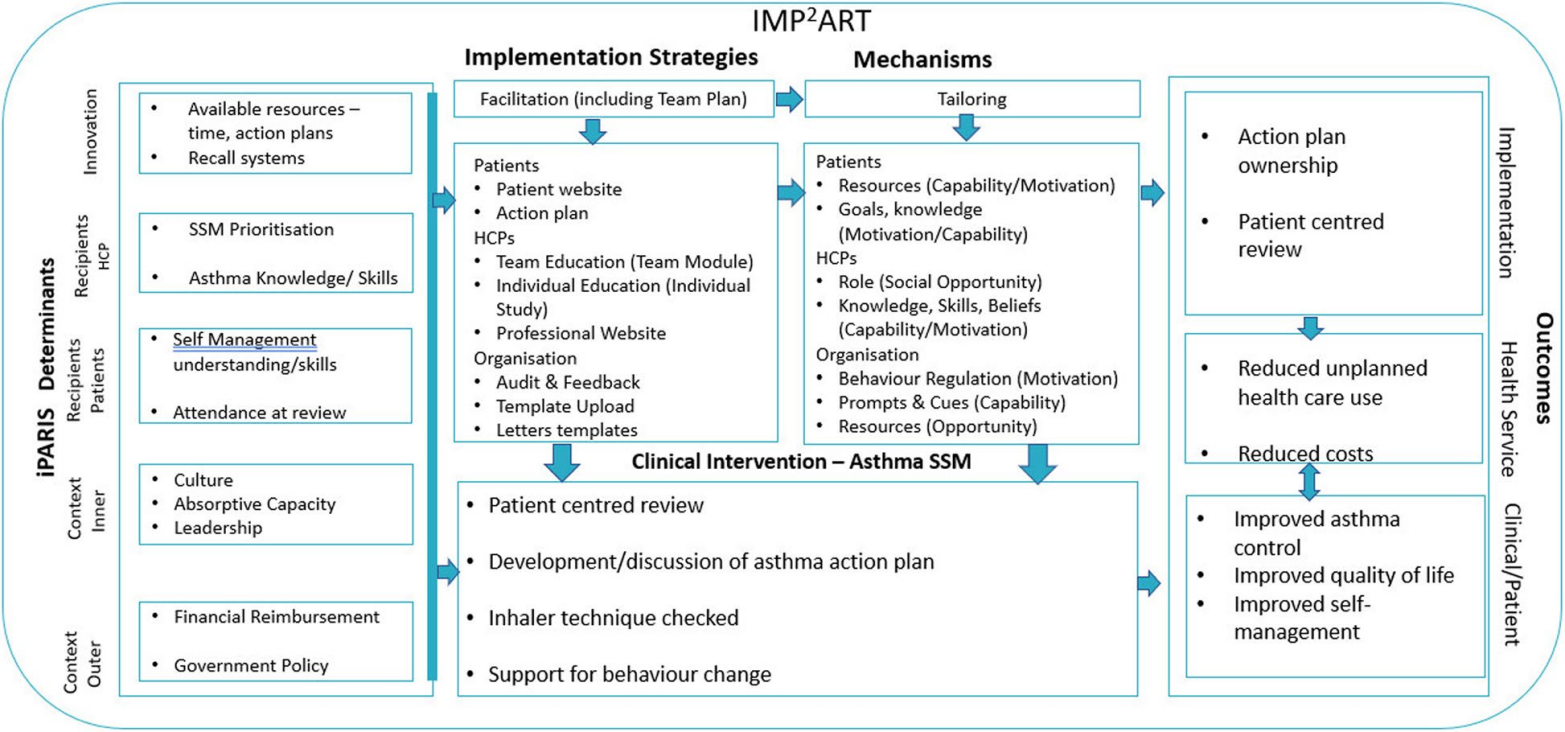
IMP2ART’s logic mode [12]. Facilitation through trained specialist asthma nurses acts as a catalyst for the use of MP2ART strategies in practices, which in turn aims to optimise professionals’ capacity, motivation and opportunity to deliver supported self-management to patients with asthma. The nature of the delivery of IMP2ART and the response to facilitation and the IMP2ART strategies will vary between practices and will be strongly influenced through practice context

The IMP^2^ART strategies comprise multiple components directed at patients, professionals and the organisation, supported by expert nurse facilitation for 12 months, summarised in a logic model (Fig. 1) and described in greater detail in McClatchey et al. (2023) [5].

While all strategies will be made available to all implementation practices, it is expected that practices will choose which IMP^2^ART strategies they use and how they adapt them to their own context. In all implementation practices, the following ‘core’ strategies will be delivered: facilitation and ongoing support from a facilitator (12 months), monthly audit and feedback reports highlighting practice-supported self-management performance (e.g. number of action plans provided) [4], and access to education modules (a team education module to highlight the whole team role in supported self-management; an individual module for clinicians delivering asthma reviews) [5] and access to a ‘Living with Asthma’ website with practice and patient resources [8].

#### Setting

The cluster randomised controlled trial takes place in England and Scotland with a target recruitment of 144 practices.

#### Pilot

An internal pilot of IMP^2^ART was conducted in 2021 with the first 12 practices recruited to the trial, primarily to optimise trial design as the feasibility of the components of the implementation strategy had already been tested and refined [4–6]. The pilot also provided an opportunity to test and refine some of the bespoke data collection and analysis approaches for the process evaluation [13].

## Methods

### Aims and objectives

The IMP^2^ART process evaluation has two primary aims:

1. To explain the IMP^2^ART trial’s clinical and implementation outcome findings
2. To identify learning, in relation to IMP^2^ART outcomes, to inform the design, scaling up and sustainability of implementation strategies to improve supported self-management of asthma in primary care

The evaluation is structured to achieve five interrelated objectives, with associated research questions, see Table 1.

**Table 1:** IMP^2^ART objectives and research questions

Objectives	Research questions
**Describe** the practice context across all IMP^2^ART practices over the course of the trial	What were the characteristics of practices recruited to IMP^2^ART?
	What is the national/local context, and how did it change over the trial?
**Measure** the extent of IMP^2^ART delivery and response (with reference to fidelity and adaptation)	To what extent was IMP^2^ART delivered as intended?
	What level of facilitation was delivered to practices?
	To what extent did practices engage with IMP^2^ART?
	To what extent was practice response as intended?
**Explore** in depth the relationship between practice context, IMP^2^ART delivery and practice response over the course of practices’ participation in the trial	What were the reasons for practices to use or engage with IMP^2^ART or not?
	What influenced the variance in IMP^2^ART delivery from what was expected?
	How did practice context interact with delivery and response to IMP^2^ART over the course of the trial?
	What were the processes by which supported self-management was implemented in practice?
**Propose explanations** for why and how the implementation strategy achieved impact (or not) with reference to the IMP^2^ART programme theory	Which conditions (implementation strategies + practice contexts) are most likely to lead to improved implementation of asthma self-management in primary care?
	To what extent does the evidence across the process evaluation support the initial IMP^2^ART theory? Is there learning from IMP^2^ART for the development or application of mid-range implementation theory?
**Provide recommendations** in relation to IMP^2^ART findings for the design, scaling up and sustainability of implementation strategies to improve supported self-management of asthma in primary care	What is the wider learning from IMP^2^ART for the design, scale up and sustainability of implementation strategies to improve supported self-management of asthma in primary care?

### Design

The process evaluation is based on the Medical Research Council guidelines for the process evaluation of complex interventions [14], also drawing on Grant et al.’s process evaluation framework for cluster randomised trials [15]. Aligned with the Medical Research Council recommendations for the evaluation at different stages of development, an internal pilot process evaluation has already been conducted and will be reported elsewhere [13]. The process evaluation incorporates learning from the pilot and focuses on three key dimensions: implementation strategy delivery (including fidelity and adaptation), practice response and practice context.

**Fig. 2:**
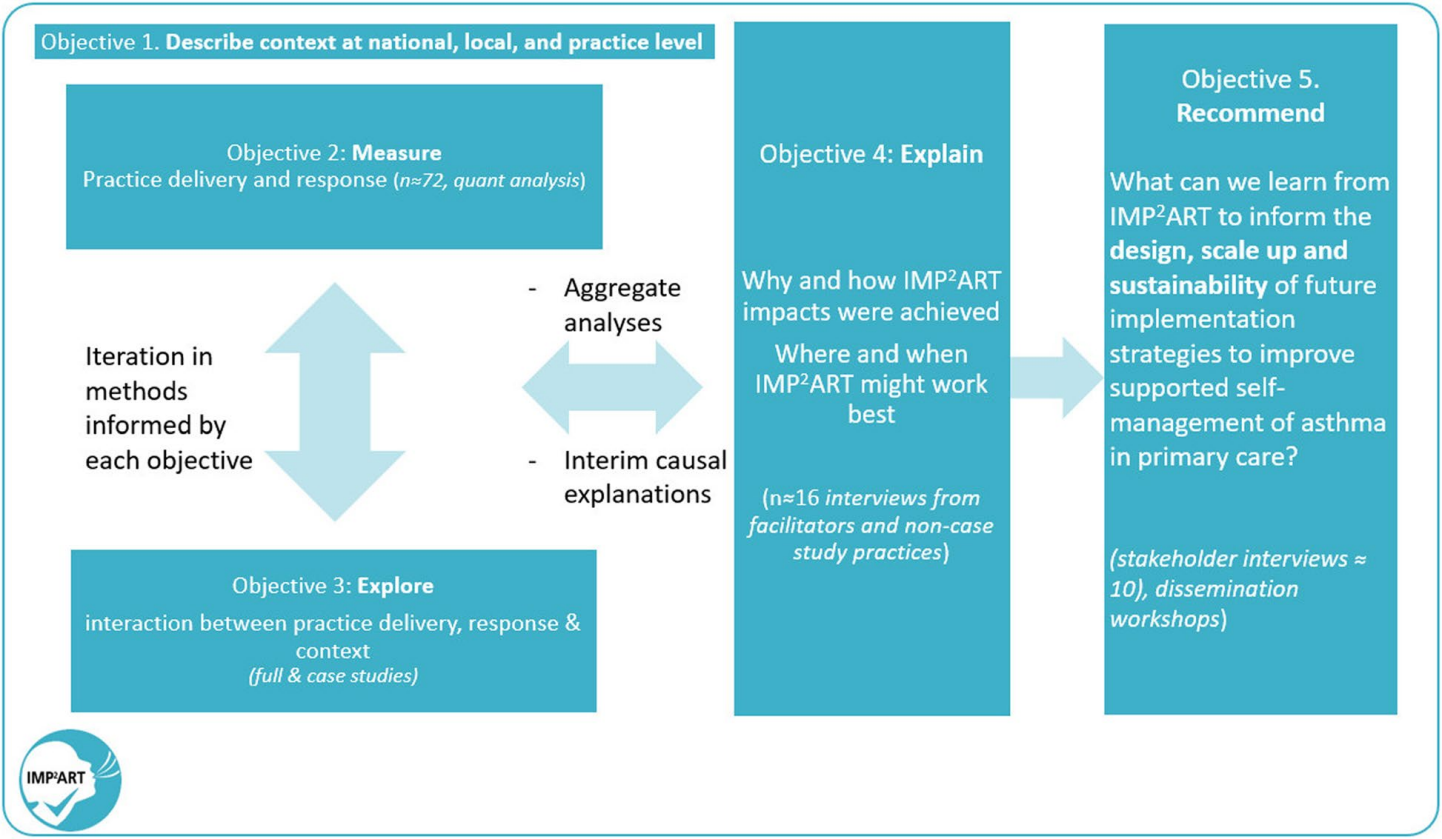
Relationship between objectives in IMP2ART’s process evaluation

As shown in Fig. 2, we will primarily use quantitative analysis to achieve objectives 1 and 2 and an in-depth qualitative approach to achieve objective 3, culminating in a mixed-methods synthesis supported by additional interviews to achieve objectives 4 and 5. In line with Fetters et al.’s description of an interactive approach, we will iterate data collection and analysis through the use of interim (formative) analyses and discussion of emerging findings during the process evaluation [16]. We will adopt a critical realist perspective, which is in keeping with our aim to derive causative explanations for IMP^2^ART’s findings, whilst acknowledging that we can only capture aspects of reality [17].

There are no specific reporting guidelines for process evaluations, so we have drawn on suggestions from Moore et al. [14] StaRI guidelines for reporting implementation studies [18] and used the TRIPLE C reporting principles for case study evaluations as a guide to the case study element [19].

### Data collection and collation

The process evaluation will use quantitative data from all 144 recruited practices (control and implementation), with a focus on the practices allocated to the implementation arm. A subset of practices will be invited to take part in further qualitative data collection, as case studies or as one-off interviews. Additional qualitative interview data will be collected from IMP^2^ART’s facilitators and national stakeholders, described in more detail below.

**Fig. 3:**
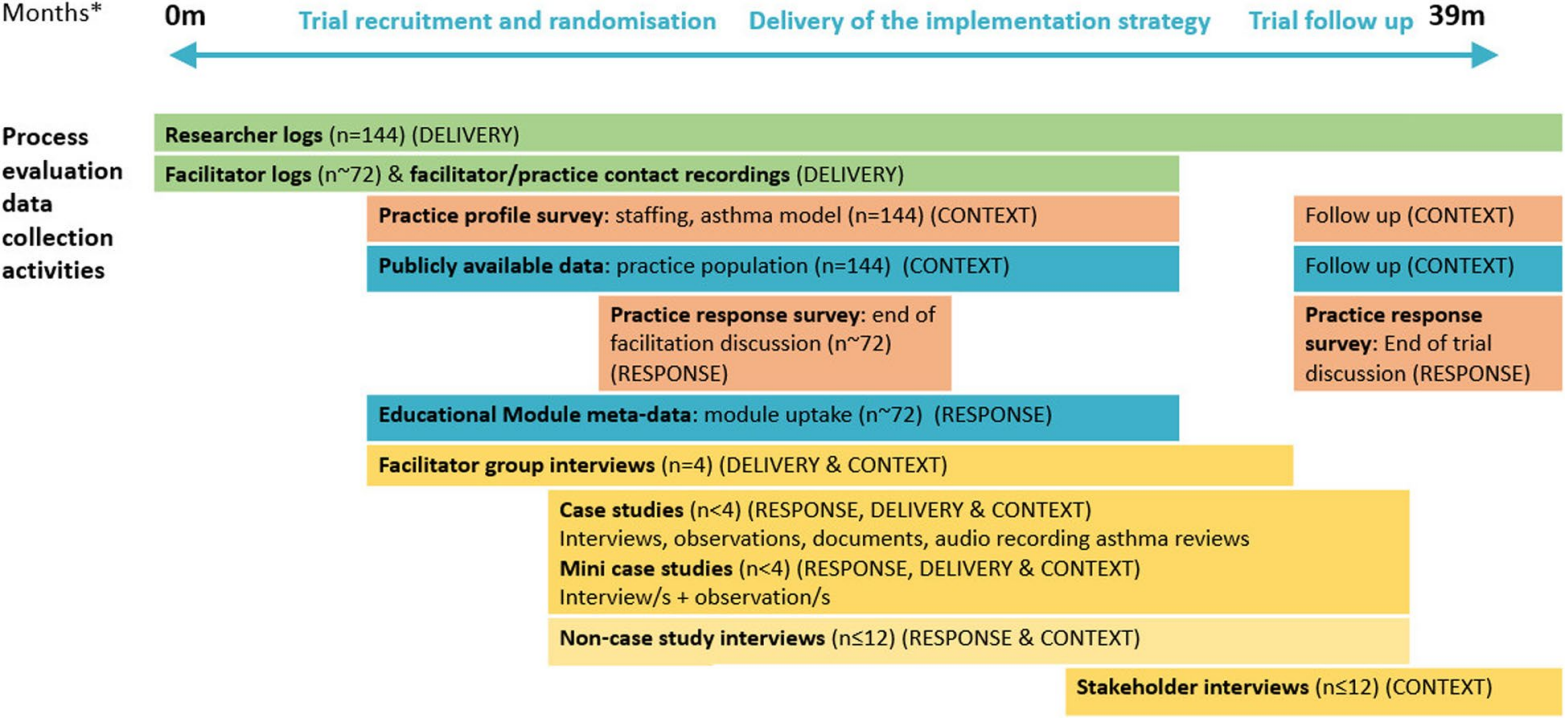
IMP2ART process evaluation data collection timeline. *Months refer to IMP2ART trial duration. All practices will participate in the trial for 24 months. Practices assigned to the implementation arm will receive 12 months of active facilitation and 12 months of follow-up

Data for the process evaluation comes from a range of sources, which are summarised in Fig. 3, and described in more detail below. Table 2 summarises their links to our research questions.

**Table 2:** IMP^2^ART data collection and analysis (*N* = 72, for all implementation practices, unless otherwise indicated)

Research questions	Data			Sources
**Practice context**				
1.1 What were the characteristics of practices recruited to IMP^2^ART? 1.2 What was the national/practice context? How did it change over the trial?	- Patient population size, age profile, ethnicity, no. of active asthma patients - Geographical region			National GP profiles [20] Census statistics IMD/SIMD CQC data^1^
	- Area-level deprivation			
	- Practice quality			
	(*n* = 144—control and implementation practices)			
	- Clinical IT system			Practice response survey
	- Self-reported training status			
	- Model of asthma care delivery (i.e. lead professional, balance of online/face-to-face asthma reviews)			
	(*n* = 144—control and implementation practices)			
	Facilitator impressions of implementation practices			Facilitator observation form based on theoretical domains framework (Additional file 1)
	Review of national and international asthma guidance 2019 vs 2024			Publicly available documents guiding supported asthma self-management (BTS/SIGN, NICE, GINA, NRAD and QOF)
**Delivery**				
2.1 To what extent was IMP^2^ART delivered as intended?	Randomisation of control and implementation practices (*n* = 144)			Researcher logs
	Time from practice agreement to completion of data review, confirming feasible to join the trial			
	Facilitator training (*n* = 4)			Facilitator + researcher logs
	• Number of training sessions			
	• Number of facilitators with successful sign-off of observed facilitation			
	• Number of supervision sessions from the lead facilitator			
	Delivery to practices—facilitation			Facilitator + researcher logs
	• % of facilitation workshops offered			
	• *N* of workshops delivered, *n* of re-scheduled/cancelled and median (range of) duration			
	• % 12-month end facilitation meeting offered			
	• % of sent team plan			
	Delivery to practices—IMP^2^ART strategies			Researcher logs
				Practice contact recordings and metadata
	• % of upload of review template			
	• Delivery of annual audit and feedback			
	• reports (3 expected over 24 months to each practice for control and implementation)			
	• Delivery of monthly audit and feedback reports (24 expected for the implementation group over 24 months)			
	• Delivery of educational module 2 logins (no practices, *n* of logins/practices)			
2.2 What level of facilitation was delivered to practices?	Number and medium of facilitator-practice interactions (minus administrative contacts)			Facilitator logs
3.1 Why did IMP^2^ART delivery vary from what was expected?	Facilitator accounts of how and why they adapted IMP^2^ART in general and in specific practices			Facilitator interviews + facilitator logs researcher logs
	Reasons documented for unanticipated changes to the delivery of IMP^2^ART by researchers			
**Response**				
2.3 To what extent did practices engage with IMP^2^ART?	Practice staff self-reported continued adoption, adaptation or discontinuation of IMP^2^ART strategies at 12 and 24 months			Practice response survey
2.4 To what extent was the practice response as intended?	- Number of workshops delivered			Practice contact recordings and metadata
	- Initial workshop date, attendance (*n* and profile)			
	- Educational modules: registration			Educational module metadata/analytics
	- Educational modules: completion of team module			
	- Educational modules: completion of individual study module			
	- End of facilitation workshop attendance (*n* and profile)			Practice contact recordings and metadata, facilitator logs
	- Practice staff self-reported use of each IMP^2^ART strategy at 12 and 24 months			Practice response survey
3.1 What were the reasons for practices to use or engage with IMP^2^ART, or not?	Practice staff perceptions and experiences of IMP^2^ART			Case studies—full and mini, plus non-case study interviews
				Selected practice response survey data, researcher and facilitator logs
	Practice feedback shared with researchers and facilitators			
**Explanations for our findings and recommendations for strategies to implement supported self-management in asthma in primary care**				
5.2 How did practice context interact with delivery and response to IMP^2^ART over the course of the trial? Which conditions (implementation strategies + practice contexts) are most likely to lead to improved implementation of asthma self-management in primary care? 5.3 To what extent does the evidence across the process evaluation support the initial IMP^2^ART theory? Is there learning from IMP^2^ART for the development or application of mid-range implementation theory? 5.4 What is the wider learning from IMP^2^ART for the design, scale up and sustainability of implementation strategies to improve supported self-management of asthma in primary care?		• Practice characteristics and uptake of IMP^2^ART • Staff views, systems and experiences of using IMP^2^ART in the context of their practice culture and routines, absorptive capacity and leadership • Facilitator reflections on adapting IMP^2^ART to the case study practices • Facilitator delivery of IMP^2^ART meetings to the case study practices • Stakeholders’ and practice staff views on the wider learning from IMP^2^ART	• Analysis of data collected for RQs 1–3 • Facilitator interviews • Non-case study interviews	

^1^England only

#### Researcher logs

Researchers will keep detailed notes about all practices approached, practices showing interest and the proportion who agree to participate. Where available, reasons for participation and non-participation will be noted to inform the potential for scaling up (*aim 2*). Reasons for withdrawal (if available) will be noted. For those practices randomised to the trial, researchers will continue to log contacts and delivery of IMP2ART tools such as audit and feedback reports sent and templates uploaded.

#### Facilitator logs and facilitator/practice contact recordings

Facilitators will keep logs of all practice contacts, including duration and nature of contact (e.g. email, phone or video call). The practice introductory workshop and the end of the facilitation meeting, both conducted via video calls, will be video recorded, and data from the call (duration, attendees and comments) will be downloaded. In addition, the facilitator will complete an observation form shortly after to structure impressions of the practice.

#### *Publicly available data and practice profile survey (n* = *144)*

Details on all trial practices (control and implementation) will be obtained where possible from publicly available sources at the start of the trial on practice characteristics (e.g. population size, ethnicity, deprivation), supplemented with a survey of all practices to obtain information on their model of asthma management. In line with StaRI, we will examine both initial and changes in context [18]. To obtain changes in the practice context, key details of this profile will be reviewed in discussion with practices at the end of the trial meeting.

#### *Educational online module usage data (n* ~ *72)*

Practice completion of the online educational modules will be logged automatically. We will store data on logins and completion of both modules at the practice level. The use of the team module will be recorded for each practice as it is designed to be used and discussed in groups.

### Practice response survey (n ~ 72)

We will collect self-reported implementation of IMP^2^ART strategies at 12 months (end of facilitation) and 24 months (end of trial). We have drawn on Proctor et al.’s taxonomy of implementation outcomes to identify different aspects of implementation relevant to IMP^2^ART and will focus on different outcomes at different points during the trial, e.g. acceptability and adoption in initial measures, adaptation at the mid-point and sustainability towards the end of the trial [21].

#### *Facilitator group interviews (n* < *8)*

IMP^2^ART’s four trained asthma nurse facilitators will be interviewed as a group at four points in the trial. Interviews will focus on their evolving experiences and learning as they progress from delivering their initial IMP^2^ART workshops and becoming experienced facilitators to their experiences of ending the facilitation process with practices. These interviews will serve a formative purpose, to provide an alert of any problems in the delivery of IMP^2^ART and to inform data collection strategies (e.g. to highlight practices that have engaged in IMP^2^ART in specific ways for non-case study interviews). They will also serve a summative purpose in providing insights into the IMP^2^ART delivery, particularly on the evolving interaction between delivery and practice context.

#### *Full case studies (n* ≤ *4) and mini-case studies (n* ≤ *4)*

We will seek to explore from multiple perspectives how IMP^2^ART fits with a practice’s culture and routines, absorptive capacity and leadership over the 2 years in which practices participate in the trial.

A case study methodology, as described by Yin, is applicable where an in-depth investigation of a contemporary phenomenon is needed within a real-life context and where the boundaries between context and the phenomenon are not clear [22]. This fits with the fact that context and intervention are intentionally very closely interlinked in IMP^2^ART because the strategies have been designed to be adapted in response to practice context.

We have designed flexibility into our case study methodology, recognising that general practices are under significant pressure and may not be able to commit to 2 years of data collection. We have therefore developed a mini-case study adaptation of our full method, as a bridge between one-off interviews and full case studies.

*Case study selection:* From the implementation group practices, we will approach case studies to try and reflect diversity in baseline asthma management and population characteristics.

*Data collection:* For full case studies, we will seek to collect several sources of bespoke data at key intervals in the practice’s participation in the trial (Table 2).

- Interviews (*n* ≤ 12/case study) with key actors will be conducted at early, mid- and later stages of the trial to track how implementation changes and changes in practice context and to explore the potential for sustainability. Key actors may be individuals who deliver supported self-management in the practice (usually nurses, but may also be healthcare assistants, pharmacists, GPs); people in an administrative role who contribute to, or might be affected by, the implementation of supported self-management (e.g. practice managers, prescribing clerks, receptionists); people making decisions about self-management (e.g. GP partners, practice managers); and lay members of a practice user group if they are involved in the IMP^2^ART initiative (see example topic guide, Supplementary data file 1).
- These will often be repeat interviews with the same individuals (or sometimes new individuals who have taken over a role of a previous participant). Researchers will tailor topic guides to the stage of the practice’s participation in IMP^2^ART, i.e. focusing on context and expectations in early interviews and asking for reflections on IMP^2^ART and sustainability at later interviews.
- Observation of activities (*n* ≤ 20 h/case), e.g. training sessions, practice meetings, facilitator visits, shadowing practice staff and routine review consultations. Observation field notes will focus on the practice context, the processes by which practices implement self-management and evidence of adopting/adapting the IMP.^2^ART implementation strategy (see example observation guide, Supplementary data file 1)
- Documentary analysis (*n* ≤ 40/case), e.g. anonymised personalised asthma action plans, meeting minutes, asthma review procedures and policies
- Audio-recording of asthma clinics *n* > 3 asthma clinics/case study practice

For mini-case studies, we will seek at least one face-to-face interview and an observation and draw on data already collected at other trial time points (e.g. end-of-facilitation meeting). Data collection will be influenced by the stage at which the practice has reached by the time of recruitment (Table 3).

**Table 3:** Dataset envisaged for case studies ✓ ✓

Data collection method	Focus	Full case	Mini-case
Observations (recordings or researcher field notes)	IMP^2^ART workshop	✓	✓
	IMP^2^ART Facilitator contacts	✓	✓
	End of facilitation meeting	✓	✓
	Practice meeting	✓✓✓	[✓]
	Practice training	✓✓✓	[✓]
	Practice environment	✓✓✓	✓
	Facilitator delivery	✓	[✓]
Interviews (recordings or field notes)	Early (e.g. expectations, adoption decisions)	✓✓	✓
	Mid-point (e.g. experiences of IMP^2^ART)	✓✓	
	Late (e.g. sustainability after facilitation)	✓✓	
Clinic recordings	Supported self-management delivery	✓✓	[✓]
Documents	Practice plan	✓	✓
	Redacted personalised asthma action plans	✓	[✓]
	Practice policies (e.g. reviews, asthma care)	✓	[✓]
	Website content review	✓	✓
Extracted from data collected on all practices	Population characteristics	✓	✓
	Practice staffing	✓	✓
	Practice asthma care delivery model	✓	✓
	IMP^2^ART audit and feedback report delivery	✓	✓
	Workshop attendance from practices	✓	✓
	Uptake of IMP^2^ART educational modules	✓	✓
	Practice-reported use of IMP^2^ART strategies	✓	✓

✓ = expected; [✓] = not essential but permitted under our ethics approval and will obtain if possible; ✓✓✓ = repeated assessment

Data collection tools are based on the IMP^2^ART programme theory, reflecting particularly the mid-range i-PARIHS and COM-B frameworks. Each tool is designed to be tailored to ensure data gathering is aligned with participants’ roles and the stage of trial participation (see Supplementary data file 1 for examples of topic guides and observation tools).

#### *Non-case study interviews (n* ≤ *12)*

Up to 12 non-case study interviews will be undertaken informed by preliminary findings from other data sources (e.g. facilitator feedback) and learning from prior IMP^2^ART research [23], so that we can explore the applicability of our emerging themes in a range of contexts. From the pool of non-case study practices, we will recruit a key informant (GP, nurse or practice manager) able to discuss the implementation of supported self-management in their practice in a semi-structured interview. Practices will be selected because they offer a contrasting context to our case study practices, use novel approaches to implementation, or are outliers in terms of outcomes/processes (e.g. where facilitator notes suggest very low or very high engagement with IMP^2^ART or exhibit innovative adaption). Interviews will be informed by the ongoing process analysis and will seek views on emerging themes.

#### *Stakeholder interviews (n* ≤ *10)*

We will arrange focussed interviews with stakeholders to explore the generalisability of emerging themes and/or policy perspectives. These may represent national or regional opinion leaders in asthma care or healthcare management with whom we can test out emerging themes. They may also include IMP^2^ART collaborators who can give a view on the feasibility and value of embedding IMP^2^ART approaches beyond the trial if they are found to be effective.

### Data management and analysis

Many of the sources will include data that could be analysed qualitatively and quantitatively. For example, the researcher logs will include dates of key activities for all practices, but for some practices, there will also be researcher field notes (e.g. practice feedback). Key variables will be extracted from the data sources and analysed quantitatively for all practices. We will also select data to be analysed qualitatively alongside case study and additional interview data to supplement the case studies or where data provide evidence that contributes to the programme theory.

### Quantitative

#### Data management

The main quantitative analysis of IMP^2^ART will be conducted at the practice level so a core dataset will be formed at the practice level from all the sources and imported into Excel.

#### Analysis

Quantitative descriptive analysis will be conducted on data from all implementation practices to answer our objective 1 research questions related to IMP^2^ART delivery, practice response and summary practice characteristics.

#### Fidelity and adaptation

The concept of fidelity has been variably defined and interpreted [24]. In some conceptualisations, fidelity is synonymous with adherence, with maximising adherence being a goal of intervention delivery [25]. In implementation research, however, adaptation of a strategy—changes in its content, format or delivery—to align the innovation with important characteristics of local context is often critical [26]. This is reflected in the StaRI guidelines which recommend reporting of both fidelity (in terms of core strategies to be delivered) and also adaptations made [18]. In IMP^2^ART, we therefore measure both fidelity of delivery for core strategies, whilst also seeking to capture adaptations and the rationale for these.

The dimensions of fidelity measured relate to the five recommended key domains of the NIH Behaviour Change Consortium (BCC) Treatment Fidelity Framework—treatment design, training, delivery, receipt and enactment [27] (with a focus on the delivery of IMP^2^ART), receipt (practice response to IMP^2^ART) and enactment (delivery of supported self-management with patients).

To allow for a more in-depth understanding of the delivery of facilitation and its potential mechanisms of action, a sub-sample of video-recorded introductory facilitation and end-of-facilitation workshops will be coded to understand the activities and processes of facilitation used. A study-specific tool has been developed for this purpose. A sample of at least 10% (*n* = 7) of practices will be selected at random stratified to ensure at least one workshop per facilitator. In addition, all workshops from case studies will be coded [28]. Each workshop will be independently coded by two individuals following training to ensure consistency in the application of the tool.

### Qualitative

#### Data management

All interviews will be audio-recorded, transcribed verbatim and anonymised. Researchers will take structured field notes for all case study observations which will be stored in a central repository. The research team will work together to build consistency in the process of both data collection and analysis. Activities to enhance consistency include all researchers watching and writing field notes on an initial IMP^2^ART workshop recording from a case study site and then discussing the similarities and differences in their observations.

#### Analysis

As advised by Yin for explanatory case studies [22], our analytic strategy for both case study data and the additional interviews will be in part guided by the theoretical propositions we have already set out, and in part inductive, guided by the data [12]. These propositions have informed the data analysis tools we have developed to organise and redescribe the data for each case. In line with critical realist theory, we also recognise the fallibility of theory and remain open to drawing on other theories or frameworks to support data interpretation [17].

**Fig. 4:**
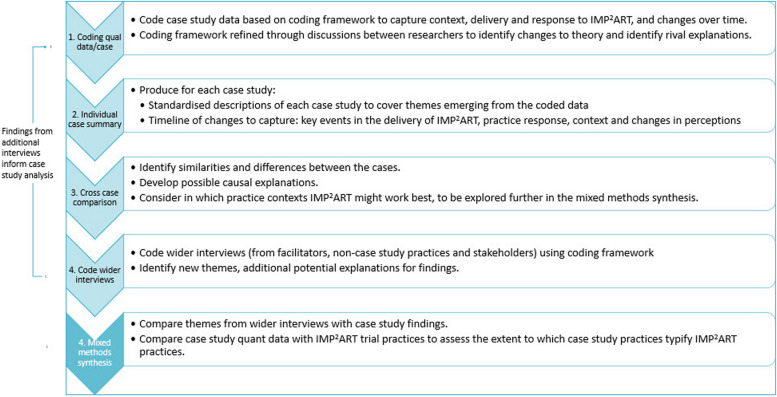
Analytical stages for qualitative and mixed methods analysis

Mid-range and programme theories have guided an initial coding framework, which we will iterate following researcher discussions in light of the data (see Fig. 4, stage 1). We will use the coded data to identify themes, and with reference to evidence from quantitative data sources (see mixed methods synthesis), we will produce both standardised descriptions of each case and a timeline. These documents will be used for cross-case comparisons to identify similarities and differences between cases (see Fig. 4, stages 2–3). As shown in step 4, wider interviews will be coded using the initial coding framework, with refinements carried out as described in step 1. The coding of these interviews will be used to identify further themes.

### Mixed methods synthesis and interpretation

In the mixed methods synthesis, we will seek to address objectives 4 and 5 providing explanation for results and making recommendations for future practice.

We will integrate quantitative data analysis (objectives 1 and 2), case study findings (objective 3) and the themes from additional interview data from group interviews with IMP^2^ART’s four facilitators; non-case study practice staff (*n* ≤ 12); and wider stakeholders (*n* ≤ 10). We will use these data to interrogate and refine the explanations derived from the case studies about which practice contexts and how IMP^2^ART might work best (objective 4).

As shown in Figs. 1 and 4, we will conduct the analysis iteratively through frequent sharing of interim analyses, to enable us to draw on learning from each data source to refine the analysis and interpretation of the data as a whole. For example, the qualitative analysis may help us select variables to examine quantitative associations between contextual characteristics and delivery/response to IMP^2^ART.

We will use integrated analysis to produce a narrative synthesis across all the data sources that critically assess the evidence for the initial programme theory [12]. If possible, we will look to understand the mechanisms and moderators that enable the use of the IMP^2^ART implementation strategies to increase supported self-management of asthma in general practice.

Preliminary findings will be shared at three end-of-programme workshops in each of the main research sites (London, Sheffield and Lothian) to share preliminary findings from the IMP^2^ART programme, gauge the application of our findings to a broader range of contexts and explore the potential for scale-up and transferability of the findings to other practices in England and Scotland.

## Discussion

### Summary

This protocol describes a nested process evaluation to aid the interpretation of the IMP^2^ART trial results and—in its own right—seeks to provide insights into the delivery and response to a whole-systems approach to the implementation of supported self-management in asthma care in general practice.

### Methodological considerations

#### Strengths

This process evaluation has been informed by a comprehensive pilot evaluation across the first twelve practices to participate in the trial and substantial research on strategies to implement supported self-management in primary care [7, 23, 29]. The pilot experience in IMP^2^ART indicated that the implementation strategy was feasible and acceptable and enabled testing and refinement of core elements of the process evaluation methods [13]. It is important to note that conditions in pilots may be different from those in the main trial [20], which necessitates exploration of some of the pilot themes with a wider sample. By conducting the process evaluation in parallel with the trial, there is the potential for interim process evaluation findings to influence peripheral content and delivery to maximise the chance of a successful outcome [28].

The inclusion of a multisite case study within the process evaluation is also now more widely recognised as a significant opportunity to provide evidence about context and transferability, and also to support elucidating causal inferences, particularly in trials like IMP^2^ART where the pathway from intervention delivery to impact is likely to be non-linear [28].

#### Limitations and challenges

IMP^2^ART is taking place at a time of considerable change and uncertainty in general practice. This uncertainty may affect both practice engagement with the implementation strategy and their participation in the process evaluation data collection. We have sought to minimise the burden for practices through using publicly available data where possible to describe characteristics and routine data for many of the measurements. Whilst maintaining consistency, we have also sought to make the process evaluation methods flexible so we can adapt to changing circumstances. For example, we have introduced an adapted case study design in recognition that many IMP^2^ART practices are not able to commit to longitudinal case studies over the entire 2 years of their trial participation. Whilst this may reduce the depth of our case studies, it will enable us to explore a wider range of contexts. We have also conducted an interim analysis of process evaluation data to identify where our methods need additional iteration.

The process evaluation is highly complex, both in terms of its combinations of theory and in its involvement of a large multidisciplinary research team split across multiple sites. Additional complexity can arise because of the differences in philosophical perspectives across the different disciplines contributing to IMP^2^ART. In order to build consistency across a large team working with these multiple perspectives, we have worked to create common understandings of all the theoretical perspectives on which IMP^2^ART is drawn [12]. We have also worked to maximise alignments between philosophical and methodological perspectives. For example, we recognise the possible philosophical misalignment between a trial, which implicitly takes a positivist perspective, and the process evaluation’s critical realist perspective, which considers that research can, at best, only capture a small part of reality [17]. Our choice of a critical realist approach, however, allows us to recognise the relevance of the trial’s findings to the reality of primary care practice and recommends the use of theory as a way of getting closer to identifying causal explanations for the trial’s findings.

It is not feasible to examine every process or outcome to the same depth. Our focus in the process evaluation is on IMP^2^ART’s main focus, helping general practices to implement supported self-management. It means, however, that while trial outcomes will be at the patient level, the process evaluation has limited opportunities to understand the impacts of IMP^2^ART on patients. We will seek to address this gap through some future additional linked projects, for example, to interview patients in some of our sites (Supplementary data file 2).

#### Study status

This protocol of the process evaluation is version 2.0. Version 1 was approved by ethics in 2019. Significant changes to the process evaluation since version 1 include the addition of adapted—mini-case studies—alongside full case studies.

Recruitment for the process evaluation began in July 2022. At the time of protocol submission (January 2024), the process evaluation was part way through the recruitment of case study sites (4/6 recruited) and had not started recruitment of non-case study interviews. Data collection is anticipated to be complete by 31 December 2024.

### Supplementary Information

Additional file 1. Data collection tools. a. Practice staff interview schedule (case study – early). b. General observation form (case study). c. Post-workshop facilitator observation form.

Additional file 2. Examples of potential allied process evaluation projects to enable additional exploration of IMP2ART’s delivery, response and context.

## Data Availability

Availability of data will depend on the data source. Due to the confidentiality of NHS routine data, trial and other data extracted from practices will not be made available. Other quantitative data may be made available via the University of Edinburgh DataShare if the practices are non-identifiable. By its nature, it will not be possible to anonymise the qualitative interview data for public availability. Requests for secondary use should be directed to the trial manager.

## Abbreviations

COM-B: Capability, Opportunity, Motivation – as determinants of Behaviour
GP: General practitioner
HS&DR: Health Service and Delivery Research
IMP^2^ART: IMPlementing IMProved Asthma self-management as RouTine
iPARIHS: Integrated-Promoting Action on Research Implementation in Health Services
NIHR: National Institute for Health Research
NHS: National Health Service
RCT: Randomised controlled trial
REC: Research Ethics Committee
UK: United Kingdom
