# Supplementary material for: Understanding the processes underpinning IMPlementing IMProved Asthma self-management as RouTine (IMP2ART) in primary care: study protocol for a process evaluation within a cluster randomised controlled implementation trial
Source: Trials. 2024 Jun 4;25:359. doi: 10.1186/s13063-024-08179-6 (PMC11151520; doi:10.1186/s13063-024-08179-6)
Supplement: Supplementary file 1 — Additional file 1. Data collection tools. a. Practice staff interview schedule (case study – early). b. General observation form (case study). c. Post-workshop facilitator observation form. [file 13063_2024_8179_MOESM1_ESM.docx]

**Supplementary data file 2**

**Data collection tool examples from the process evaluation**

1. **Interview schedule**
2. **General observation form**
3. **IMP2ART workshop observation form**
4. **Interview schedule**

**To be used with Healthcare Professionals employed in IMP2ART case study practices
August 2023 v2.1**

*Notes for researchers:*

- *Feel free to re-phrase the question to ask about this concept in your own words.*
- *The interview guide is generic for any member of staff. You will need to tailor the guide to ensure questions are appropriate to the role of the participant in the practice. Use Section 1 to help guide this.*
- *Feel free to re-order as you need to make the interview flow*
- *If remote, could check in on where are you doing the interview? Shared space? Work? Home?*
- *For early and late interview: share the culture competing values framework*
- *Have the practice plan to hand (consider sharing in advance)*

**TIMEPOINT: EARLY MID LATE**

*Case study practice staff could have up to 4 interviews over the course of the whole trial.*

- *Early ~ 0-3 m after IMP2ART workshop*
- *Mid ~ 9-12m after workshop*
- *Late ~ 15-24 month after workshop*

*The questions will change depending on the stage of the trial.*

Thank you for agreeing to this interview. As a case study practice, in addition to learning about your experience of IMP2ART, we are also hoping to understand your practice context in more depth.

We look forward to speaking to you or your colleagues at 2-3 other points during the trial to explore any changes in your practice and in your experience of IMP2ART.

| **Role in the practice in general & in asthma care** |
| --- |
| Possible prompts   - Leadership role in practice/beyond? - Involvement/responsibility for asthma reviews? - How is asthma care documented in the practice/information shared between practice HCPs? - Changes in your role over time? |
| **Experience/perspective on delivering supported self-management in asthma.** |
| We’d like to explore the understanding of the term supported self-management, normal delivery in your practice.  Views on possible barriers to delivering supported self-management  Probe   - Patient engagement/uptake e.g. attendance at asthma clinics - Staff factors e.g. access to/uptake of relevant training, teamwork, healthcare professionals’ behaviours including skills/capability - Alignment of practice routines, opportunities to deliver SSM and patient needs e.g. discussion/management of multimorbidity - External pressures or support   Probe: explore how these factors might limit/ constrain SSM?  Probe: individual changes, practice changes, QOF? Covid? Remote reviews? |
| **Practice context** |
| We are interested to understand how IMP2ART works in different practices, so we’d like to understand a bit more about your practice. I’d like to start by asking you about your views on the way things are done in your practice and to understand its values.   - 1. Some people characterize culture (general beliefs, values, assumptions that people embrace) in terms of four general types. We’d now like to ask you to look at the image shared with you before this interview [*researcher: show the interviewee as shared screen or show a print out],*   **Which one or ones do you think your practice conforms most to, and why?**   1. How do you typically find out about new information, such as new initiatives, accomplishments, issues, new staff, staff departures within the practice? 2. Can you tell us about a recent QI project that has happened in the practice?   *Possible prompts: how does your practice normally respond/manage change support for quality improvement initiatives in and beyond the practice, e.g. federation, CCG, other? Any dissemination activities within or beyond the practice?*   1. What support do you get [from the practice] for learning new skills?   *Possible prompts: Protected time, budget for training, encouragement to attend*   1. Can you give an example of where a recent problem was identified in the practice, how it was recognised and resolved? OR…*If they can’t think of an example:* If you saw a problem in your own setting, what would you do?   Some prompts:   - - To what extent do **you** feel like you can try new things to improve your work processes?   - Do you feel like you have the **time and energy** to think about ways to improve things?   - Would you **trust** your practice (direct line manager) would **support** you in trying something new, even if it didn’t work?   **Prior use/experience of strategies for quality improvement in asthma care?** *Audit & feedback, templates, staff training, patient template letter* |
| **Expectations of IMP2ART** |
| Your practice is in its first months of IMP2ART. I’d like to ask you first about the decision to take part and then about **your expectations** for the next two years.  Can you tell us about how you first heard about the possibility of taking part in IMP2ART?  [*tailor according to role: lead/decision maker vs nurse vs administrator*] What influenced your decision to take part in IMP2ART? OR…What do you think influenced your practice’s decision to take part?  How do you think IMP2ART fits (or not) with your practice’s culture/ethos?  *[Explore the following if not mentioned unprompted]*:   - alignment with practice goals, e.g. importance of asthma to practice; needs and preferences of patients; other practices were getting involved; local, or national performance measures, incentives (e.g. QOF), policies, regulations, or guidelines; importance of a whole team approach.   How does Imp2ART fit with your own priorities and capacity?  *Possible prompts: existing skill set; time to do the training/consider changes to practice*  **How do you think IMP2ART might change the ways you work with the rest of the team?**  Possible prompts:  Effects on who does what?  Effects on ease and effectiveness of consultations?  Skills gaps for you or for others/how or whether they will be addressed?  What is the general level of receptivity in your practice to implementing IMP2ART?  prompt: How do you think your **practice's** **culture** will affect the implementation of IMP2ART?  *[tailor to individual being interviewed]* What kind of support or actions do you think you will need to provide as a leader in your practice? OR….What kind of support or actions do you expect from **leaders in your practice** to help make implementation successful? *Possible prompts: How do attitudes of different leaders vary? What types of barriers might they create? [SUPPORTIVE LEADERSHIP]*  **Patient engagement**  How do you feel **patients** might respond (*or are already responding if appropriate*) to changes in self-management (probes for delivery – patient centred consultation – template guided, Action plans, letters, website; remote review guidance) – e.g. effect on access, engagement in asthma reviews? Motivation/capacity to self-manage? |
| **EXPERIENCE OF IMP2ART SO FAR [FACILITATION]** |
| Did you attend the IMP^2^ART workshop?  Q: What was your experience of this?  Possible prompts:   - What did you think you/the practice gained from it? - What didn’t work or could have been improved? - How do you think will enable your practice /you to improve delivery of SSM with asthma patients?   What did you see as the role of the IMP2ART facilitator? Prompt: can you recall any processes the facilitator went through?  **Your practice agreed a practice plan as a result of the workshop** *[share their plan as screen share]*  Possible prompts:   - How did you arrive at x in the plan? - What do you see as your role in helping the practice to achieve x?   Q: What else have the facilitators done? [*note for researchers – explore unprompted first, then could give examples from this list* *rather than going through it all systematically.***]**   - **Professional resources** - Team education module - Individual education module - **Patient resources** - Action plan templates - On-line information resources (<http://livingwithasthma.org.uk>   - Links to information on asthma/asthma treatment   - Links to asthma-relevant COVID information   - New enhancing IMP^2^ART resources (Media Clips/infographics)   - New remote resources (Patient-facing aspects of tool kit) - Invitation letters - Waiting room posters - **Organisational strategies** - Review templates - Annual audit and feedback report - Monthly audit and feedback e-mails, top tips - Online ‘review’ questionnaire and workflow   In what ways have these supported you in delivering SSM?  Turning your thoughts to internal facilitation, has anyone in your practice taken on the role of facilitator or leader for IMP2ART in the practice? How was that decided? |
| **Thanks and final points** |
| Anything else you would like to share? |

1. **IMP^2^ART practice observation guide**

**To be completed for every observation of the practice.**

**Final Version 1.2 June 2022**

1. Purpose and guidance:

This guide is intended to help structure and standardise your observations and prompt reflections when you are undertaking observations in the practice reviews. The intention of these observations is twofold:

1. enactment of IM^2^PART strategies e.g. presence of posters, staff undertaking processes of inviting patients for review, meetings to review IMP^2^ART progress with/without facilitator
2. aspects of the practice context, including staff motivation, opportunity and capability, practice culture, including:

- **Leadership** (e.g. formal and informal leadership styles, such as degree and type of supervision, degree of support and trust, degree of aloofness, and type of leadership hierarchy)
- **Staff/team responsibilities, behaviours and relationships** (characteristics of interpersonal interactions, group behaviours, perceptions of co-worker trust, degree of group supportiveness, group cohesion, and coordination of group effort),
- **Patient centeredness** (in general and in relation to asthma
- **Communication** (formal and informal)
- **Practice population**
- **Governance** and performance management arrangements
- Availability of **material resources** (e.g. equipment, premises)

*A reminder when you arrive: announce your presence and ensure you have on an identification badge if you are observing in person.*

1. Essential details:

| **Case study site** |  |
| --- | --- |
| **Activity being observed** | Training/Meeting/Shadowing/Other (delete as needed) |
| **IMP^2^ART specific?** | Yes / No |
| **Remote/in person** |  |
| **Date** |  |
| **Researcher name** |  |
| **Duration of observation** |  |

1. Prompts for things to look for/make notes on:

Some signs to look out for are listed below but you may well notice others:

| **Physical Environment and objects**  • Where is the meeting or activity taking place?  • What is the space like?  o Cluttered/Neat? Quiet/Loud? Crowded/empty? Bright/Dim? Other?  o Who sits or stands where relative to others?  • What is signage or materials on the walls like?  o Up-to-date? In good condition?  o Anything asthma related?  **Timing/structures**  • What is the frequency and duration of the meeting/rounds you are observing?  • Is this a new or established processes? | **Actors/Participant behaviours**  • Who is doing what?  • Who interacts with whom?  How do participants address each other/patients? Formality?  o Who empowers or silences whom?  How is power and authority exercised?  • Who is missing?  **Informal factors**  • What non-verbal cues do you observe? (e.g. eye contact, folded arms, checking phones)  • What other visual cues do you observe?  **Nature and** **content of discussion**  • Topics discussed  • Any issues causing conflict |
| --- | --- |

*Tips for giving evidence of impressions:*

- put a quote and add your reflections (e.g. XXX quote exemplifies an interpretation of a practice as patient centred/non patient centred.)

1. Next steps

What further information do you think might be useful to collect to understand the practice better? E.g. Are there questions that have arisen for you in how the practice works/how key people work together? Are there ideas that you think should be tested /developed?

Consider **documents** (e.g. policy or procedure that was mentioned), **participants** for interview, **activities** for future observations

1. **Obtaining rapid insights into the practice context: observation form**

**26 April 2023**

**V 3.0**

- Complete as soon as possible **AFTER** the workshop
- **You don’t need to complete every section – it is OK to leave any parts blank if it wasn’t possible to form an impression**

| 1. **Individual staff attendance & participation** | Attendance | Contribution to discussion | | | | | Informal or formal leader? | |
| --- | --- | --- | --- | --- | --- | --- | --- | --- |
|  |  | Not at all | Minimal | Some participation | Led discussion | Dominated discussion | in the practice  Yes/no/somewhat | Asthma SSM  Yes/no/somewhat |
| Nurse |  |  | | | | |  | |
| GP - partner |  |  | | | | |  | |
| GP – salaried or locum |  |  | | | | |  | |
| Other healthcare professional |  |  | | | | |  | |
| Non clinical - manager |  |  | | | | |  | |
| Non clinical – administrator |  |  | | | | |  | |
| Other |  |  | | | | |  | |

| 1. **Impressions of the practice culture** | | Strongly disagree | Disagree | No opinion | Agree | Strongly agree |
| --- | --- | --- | --- | --- | --- | --- |
|  | This practice culture seems primarily **collaborative** (eg all are invited/confident to contribute to discussion and decision making) |  |  |  |  |  |
|  | [*From the discussion on OPC report*], this practice appears **open to new information** about itself |  |  |  |  |  |
|  | [*From the discussion in forming the practice plan*] this practice appears to have the (collective) **capacity to form achievable plans** |  |  |  |  |  |
|  | [From the discussion] this practice appears to be **willing** to make improvements to its procedures to improve patient care |  |  |  |  |  |
|  | [From the discussion] this practice appears **capable** to carry out improvements to its procedures to improve patient care |  |  |  |  |  |

| 1. **Impressions of practice/team knowledge and capacity** | Strongly disagree | Disagree | No opinion | Agree | Strongly agree |  | Confidence in Rating on a scale of 1-10  1-not at all 10 - completely |
| --- | --- | --- | --- | --- | --- | --- | --- |
| Knowledge is sufficient for implementing SSM |  |  |  |  |  |  |  |
| The team have the skills for SSM |  |  |  |  |  |  |  |
| The team see SSM as part of their roles |  |  |  |  |  |  |  |
| The team have the confidence to implement SSM |  |  |  |  |  |  |  |
| The team are positive about implementing SSM |  |  |  |  |  |  |  |
| The team need reinforcement (financial or social) to implement SSM |  |  |  |  |  |  |  |
| The team intend to implement SSM |  |  |  |  |  |  |  |
| The team have clear goals to implement SSM |  |  |  |  |  |  |  |
| The team will remember to implement SSM |  |  |  |  |  |  |  |
| The team have the resources to implement SSM |  |  |  |  |  |  |  |
| Social influences support SSM |  |  |  |  |  |  |  |
| Team morale will help implement SSM |  |  |  |  |  |  |  |
| The practice know is they are implementing SSM |  |  |  |  |  |  |  |

Free-text notes – please use this space to capture anything about the process or the practice that you think might inform how you will tailor IMP2ART for this practice or how this practice might respond to IMP2ART. This might include:

- Experience of innovation & change – any discussion of successes in improvements they have made in the past, whether they give examples of being research active
- Views on their current performance in asthma/self mgt or general

questions that arose for you about the practice and its readiness to implement changes to its asthma SSM practices that you’d like to explore in future interactions
