# Supplementary material for: Understanding the processes underpinning IMPlementing IMProved Asthma self-management as RouTine (IMP2ART) in primary care: study protocol for a process evaluation within a cluster randomised controlled implementation trial
Source: Trials. 2024 Jun 4;25:359. doi: 10.1186/s13063-024-08179-6 (PMC11151520; doi:10.1186/s13063-024-08179-6)
Supplement: Supplementary file 2 — Additional file 2. Examples of potential allied process evaluation projects to enable additional exploration of IMP2ART’s delivery, response and context. [file 13063_2024_8179_MOESM2_ESM.docx]

Supplementary data file 3.

**Examples of potential allied process evaluation projects to enable additional** **exploration of IMP2ART’s delivery, response and context**

| **Project** | **Data source and proposed analysis** |
| --- | --- |
| Indepth analysis of practice plans formed during the Imp2ART workshop | Analysis up to 72 practice plans developed during the IMP2ART workshop to understand practice-identified priorities to develop supported self-management for their asthma patients. |
| Qualitative understanding of patient experience of supported self-management in IMP2ART practices | Ethics permission is being sought to recruit up to 15 (male and female) patients who have a diagnosis of asthma and attend their general practice for their asthma reviews |
| Uptake, response and completion of educational modules | For the individual clinical educational module, data will be available on the number of team members completing the module, along with the choices that participants make where options are provided (e.g. of exploring a range of different barriers to providing self-management support). These data will be used for an allied project to provide a more indepth understanding of the educational module completion and response by practice staff. |
